# Risk of Cardiovascular and Cerebrovascular Events in Polycystic Ovarian Syndrome Women: A Meta-Analysis of Cohort Studies

**DOI:** 10.3389/fcvm.2020.552421

**Published:** 2020-11-12

**Authors:** Jun Zhang, Ji-Hong Xu, Qian-Qin Qu, Guo-Qing Zhong

**Affiliations:** ^1^Department of Obstetrics and Gynecology, The First People's Hospital of Jiangxia District Wuhan City, Wuhan, China; ^2^Department of Outpatient, Taihe Hospital, Hubei University of Medicine, Shiyan, China; ^3^Department of Obstetrics, Zhuxi People's Hospital, Zhuxi, China; ^4^Department of Obstetrical, Jining No.1 People's Hospital, Jining, China

**Keywords:** polycystic ovary syndrome, cardiovascular disease, myocardial infarction, stroke, mortality

## Abstract

**Aim:** This systematic review and meta-analysis aimed to investigate the risk of cardiovascular disease (CVD) and cerebrovascular disease (CeVD) events in women with polycystic ovary syndrome (PCOS).

**Methods:** We searched the literatures in Pubmed, Embase, and Web of Science to identify cohort studies reporting the association between PCOS and CVD/CeVD events from 1964 to June 1, 2020. Outcome variables, such as all-cause mortality, cardiovascular death, any cardiovascular diseases, myocardial infarction, ischemic heart disease, and stroke, were extracted from the identified literatures, and we reported the outcomes of the association in hazard ratios (HR) and odds ratios (OR).

**Results:** Ten cohort studies comprising 166,682 samples are included in the review. Compared to non-PCOS women, the pooled risk of CVD events in PCOS women (OR: 1.66, 95% CI: 1.32–2.08). In addition, the risk of myocardial infarction (OR: 2.57, 95% CI: 1.37–4.82), ischemic heart disease (OR: 2.77, 95% CI: 2.12–3.61), and stroke (OR: 1.96, 95% CI: 1.56–2.47) are higher in the PCOS group. However, no significant difference in the overall mortality (HR: 1.04, 95% CI: 0.57–1.86) and CVD-related death (HR: 1.49, 95% CI: 0.99–2.23) was observed. Funnel plots of all outcomes are roughly symmetric, and no significant publication bias was found.

**Conclusion:** Though this study identified an increased risk of CVD and CeVD among women with PCOS, including occurrence of myocardial infarction, ischemic heart disease, and stroke, there was no difference in the all-cause or CVD-related mortality observed. Further large-scale studies are warranted to strengthen the association between PCOS and CV events. Our study may require a larger sample size to further verify the conclusions.

## Background

Polycystic ovary syndrome (PCOS) is an endocrine disease, which shows common polymorphic and clinical manifestations and 5–10% of morbidity in women of reproductive age (1, 2). It is caused by follicular dysplasia, insulin resistance (IR), and hyperandrogenism, which is characterized by irregular menstruation, infertility, obesity, and hyperandrogenemia (3, 4). PCOS patients often experience metabolic disturbances, which are easily combined with lipid metabolism disorders, obesity hypertension, diabetes, and other metabolic synthesis (5). Thus, PCOS patients have increased risk of atherosclerosis, which thereby significantly increases the risk of cardiovascular disease (CVD) (5).

CVDs are the number one cause of death globally, taking an estimated 17.9 million lives each year. CVDs are a group of disorders of the heart and blood vessels and include coronary heart disease (CHD), cerebrovascular disease (CeVD), rheumatic heart disease, and other conditions. Four out of five CVD deaths are due to heart attacks and strokes, and one third of these deaths occur prematurely in people under 70 years of age (6). PCOS patients are often associated with obesity; most patients show a tendency to be obese during or even before puberty, and obesity is a risk factor of CVD, which thereby increases its incidence (7). Abnormal lipid metabolism often exists in PCOS patients with an approximately 70% prevalence rate, and it is mainly manifested as high-density lipoprotein (HDL) decrease and low-density lipoprotein (LDL) increase (8). At present, there are various studies on the risk of PCOS and CVD, but the results differ. Meta-analyses (9, 10) suggest that PCOS patients have a higher risk of CVD than normal people. In the study published in 2016, the results showed that a significant association was found between PCOS and CHD, no significant association was observed between PCOS and myocardial infarction (MI). In an article published in 2017, compared with those without PCOS, subjects with PCOS were significantly associated with an increased risk of developing stroke. However, no significant association was observed between PCOS and all-cause death in that article. In addition, a review of longitudinal studies (11) on the increased CVD prevalence is not proven. Therefore, we performed this meta-analysis to better clarify the relationship between the PCOS and the risk of cardiovascular and cerebrovascular events and mortality.

## Materials and Methods

### Search Strategy

Our search used the guidelines of Preferred Reporting Items for Meta-analysis. We obtained a list of eligible studies from the following databases: PubMed, EMBase, and Web of Science, published in English up to June 1, 2020. The search MESH term and keywords used included “polycystic ovary syndrome,” “PCOS,” “Stein-Leventhal syndrome,” “sclerocystic ovarian degeneration,” “cardiovascular diseases,” “coronary heart disease,” “myocardial infarction,” “cardiovascular stroke,” “myocardial infarct,” “heart attack,” “ischemic heart disease,” “myocardial ischemia,” “stroke,” “cerebrovascular accident,” and “apoplexy.” Detailed search strategies are shown in Supplementary Method 1.

### Inclusion and Exclusion Criteria

The inclusion criteria for this study were (1) exposed group was defined as PCOS according to the PCOS diagnostic criteria. Diagnosis of PCOS was classified as definite and possible PCOS. Definite PCOS was defined as histological evidence with clinical evidence of ovarian dysfunction. Possible PCOS was defined as histological evidence with clinical information not available, macroscopic evidence with clinical evidence of ovarian dysfunction, or clinical diagnosis by an experienced consultant (12). (2) The non-exposed group was defined as those without PCOS disease. (3) Cardiovascular outcomes included subclinical and clinical cardiovascular disease prevalence, incidence, and mortality. The outcomes were defined as mortality rate, which includes all-cause mortality and cardiovascular death, and cardiovascular event incidence, including any CVD, MI, ischemic heart disease, and stroke. Cardiovascular death was defined as that resulting from sudden cardiac death, end-stage congestive heart failure, acute MI, peripheral arterial disease, or cerebrovascular accident. (4) The study design was cohort studies only, and the language of the included stuies was limited to English.

The exclusion criteria for this study were (1) the patient had a prior cardiovascular event; (2) the study was a conference, abstract, or letter; (3) republished studies; (4) outcome data of study were unavailable.

### Data Collection and Quality Assessment

Relative data were extracted by two independent authors (JZ and CG) with a unified standard. Differences or contradictions between the authors were resolved by discussion or consultation of a third investigator (GQZ). We extracted relevant information from the included studies, such as (1) basic characteristics of individual study: first author, published year, country, mean age, sample, study design, race, PCOS diagnosis, control group, adjustment factors, and follow-up; (2) clinical physiological and biochemical characteristics of individual study: BMI, waist-to-hip ratio, smoking rate, postmenopausal women, systolic, diastolic, hypertension, diabetes, IR, HOMA dyslipidemia, total cholesterol, LDL and HDL cholesterol, triglycerides, androstenedione, testosterone, free testosterone, and fasting glucose; (3) outcomes: all-cause mortality, cardiovascular death, any CVD, MI, ischemic heart disease, and stroke. In the original literature, if there is original data, the data on the original event is extracted; if not, the combined effect quantity is extracted. If multiple effect estimates are reported, we use the most comprehensive adjusted risk estimates.

Methodological quality of the studies was assessed using the Newcastle-Ottawa Scale (NOS) with eight items. A study can be rewarded a maximum of nine stars with a maximum of two stars for comparability and one star for each numbered item within the selection and exposure categories. More than six stars indicates a study of high quality.

### Statistical Analysis

The effect size of hazard ratio (HR) (13) with 95% confidence interval (CI) was used for the outcome of mortality rate, and the odds ratios (OR) (14, 15) with 95% CI was used for the incidence of cardiovascular and cerebrovascular events. The *I*^2^ statistical value using chi-square tests was used to evaluate and measure the heterogeneity size (16). When the I^2^ value was >40% with significance level of *P* < 0.1, heterogeneity was identified in relevant outcome. Funnel plots were used to qualitatively detect publication bias (17). Statistical analyses of all outcomes were performed using RevMan software (Versions 5.3).

## Result

### Literature Search Results

**Figure 1:**
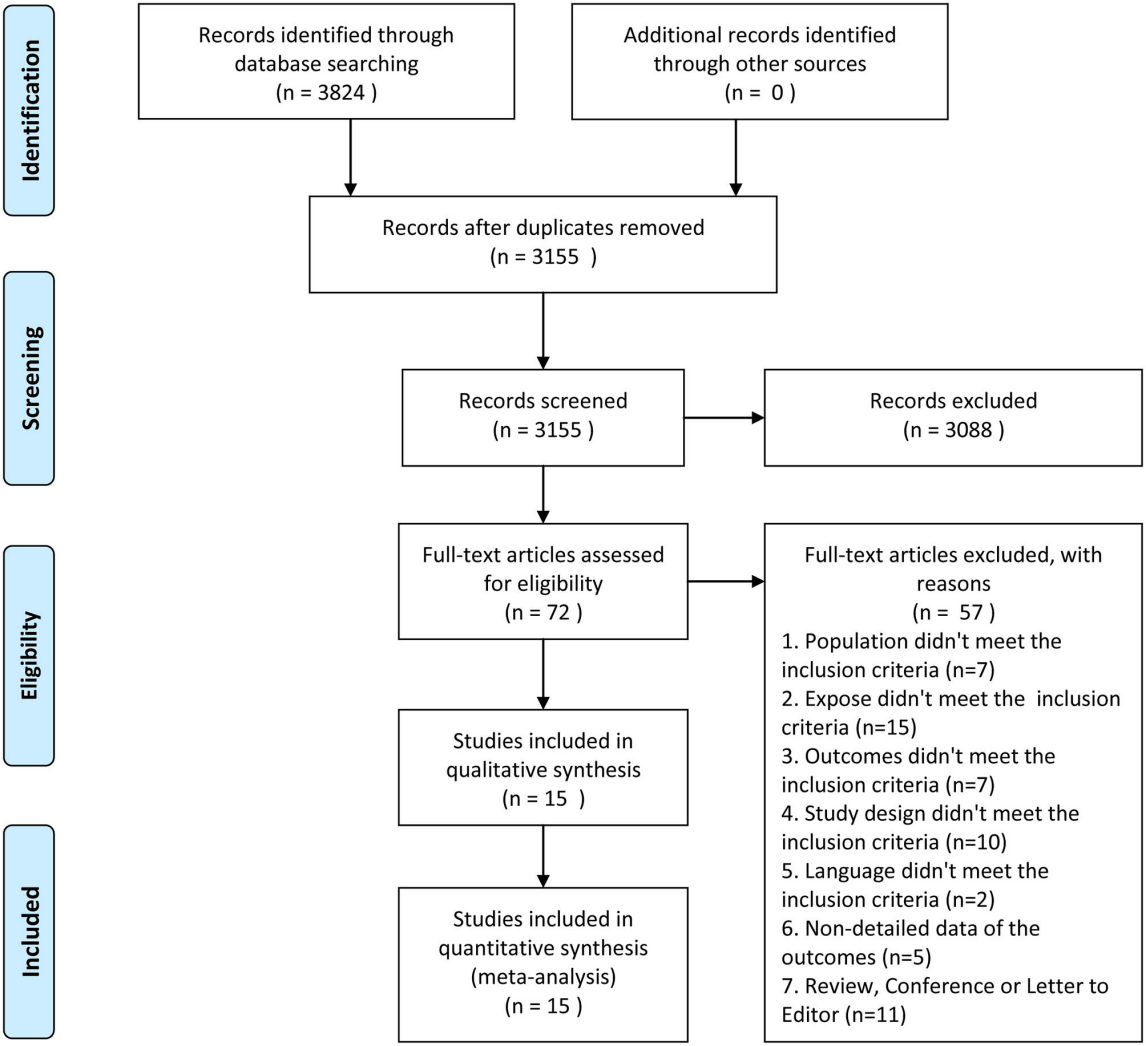
Flow diagram of selection process.

Initially, 3,824 literatures were identified from PubMed, EMBase, and Web of Science. However, 669 of those were excluded due to duplication, 3,088 studies were excluded by reading titles and abstracts based on the strict inclusion and exclusion criteria, and 72 potential studies were screened for full-text reading. Finally, 15 cohort studies with 352,031 individuals (18–32) were chosen (Figure 1). Average follow-up duration ranged from 5 to 22 years. Patients were followed up for an average of more than 10 years in a majority of studies (73.3%). Ten of the articles' risk estimates

were adjusted for age and BMI. Ten studies (66.7%) reported an adjusted estimate for at least one of the risk factors. The basic characteristics of the included studies are shown in Table 1, and the clinical physiological and biochemical characteristics of individual studies are provided in Table 2.

**Table 1:** Characteristics of individual studies.

**Study**	**Year**	**Country**	**Age**	**Sample**	**Race**	**Diagnosis of PCOS**	**Source of control**	**Adjustment factors**	**Follow-up (years)**
Calderon-Margalit	2014	USA	45.4(3.44); 45.4 (3.57)	55/668	Black 376, White 374	NR	Women who had none of these symptoms	Age, Race, Education, Smoking, Menopausal Status, BMI, SBP, lnTG, and HOMA-IR	20
Dahlgren	1992	Sweden	45.9(40-49); 55.1(50–61)	40–49 Y: 18/57; 50–61 Y: 15/75	NR	Histopathological characteristics	Age matched referents	NR	12
Ding	2018	Taiwan	15–49	8,048/32,192	NR	ICD-9	NR	NR	16
Glintborg	2015	Denmark	29(23–35)	77,899	NR	ICD-10	The index date of their matched PCOS cases	NR	17
Hart	2015	Australia	NR	2,560/26,660	NR	ICD-10 or ICD-9	Hospital population	Any potential effect of PCOS on hospitalizations related to adult onset diabetes	15
Iftikhar	2012	USA	25.0 ± 5.3	309/652	NR	ICD	Matched age and calendar year during their clinic visit plus three years	Age at last follow-up, BMI, infertility treatment, postmenopausal hormone therapy, and family history.	22
Joan	2006	USA	30.7 ± 7.2/30.8 ± 7.5	11,035/55,175	White 1,209/3,778, Black 226/522, Asian 288/1,117, Hispanic 352/1,324, Other 140/432	ICD-9	Health plan membership	Hypertension, dyslipidemia, diabetes mellitus, and BMI	NR
Lunde	2007	Norway	49.8(42.8, 57.4)/46.6(35.6, 57.2)	131/854	NR	Ultrasound examination and histological examination	Matched age, birth cohort and observation time	NR	15–20
Mani	2013	UK	29.6 ± 9.1	2,301	White 1,479, South Asian 677, Black 26, Other 119	Clinical and biochemical grounds	National Female Population	BMI, age and hypertension	20
Merz	2016	USA	62.6 ± 11.6/64.8 ± 9.6	25/270	NR	1990 NIH criteria, 2003 European and American criteria	Registered menopausal population in the WISE	Diabetes, waist circumference, hypertension, and angiographic CAD	10
Morgan	2012	UK	27.1 ± 7.1/27.1 ± 7.1	21,734/108,670	NR	NR	Matched primary-care practice, age and BMI	Age, primary-care contacts, BMI, and year of diagnosis	5
Meun	2018	Netherlands	69.57 ± 8.72/69.20 ± 8.60	106/171	NR	NR	NR	Age, years since menopause and cohort	12
Schmidt	2011	Sweden	NR	32/127	Caucasian	Rotterdam criteria	Age-matched control	NR	21
Shaw	2008	UA	62.5 ± 10/65.8 ± 9	104/390	NR	NR	Women without clinical features of PCOS from NIH-NHLBI	Age and body mass index	6
Wild	2000	UK	NR	319/1,379	NR	Histopathology records, operating theater records, admission and discharge records and diagnostic indexes	Age matched referents	BMI	5

*BMI, body mass index; ICD, International Classification of Diseases; NR, Not reported; PCOS, Polycystic ovary syndrome*.

**Table 2:** Clinical physiological and biochemical characteristics of individual studies.

**Study**	**Year**	**BMI**	**Wraist to hip ratio**	**Smoking rate**	**Post-menopausal women**	**Systolic (mmHg)**	**Diastolic (mmHg)**	**Hypertension**	**Diabetes**	**Fasting glucose (mg/dl)**
Calderon-Margalit	2014	29.3 (6.50);29.9 (7.47)	NR	23/287	7/153	113 ± 15.9/115 ± 16.3	71.6 ± 11.8/72.7 ± 11.7	10/118	4/36	100 ± 36.3/95.4 ± 22.4
Dahlgren	1992	NR	40–49 Y: 0.81 ± 0.06/0.78 ± 0.06; 50–61 Y: 0.84 ± 0.09/0.79 ± 0.07	NR	NR	NR	NR	40–49 Y: 18/75; 50–61 Y: 15/90	40–49Y: 18/75; 50–61 Y: 15/90	NR
Ding	2018	NR	NR	NR	NR	NR	NR	NR	NR	NR
Glintborg	2015	27.3(23–32.7)	NR	51/82	NR	NR	NR	357/365	458/423	4.6(4.3–5.0)
Hart	2015	NR	NR	NR	NR	NR	NR	NR	NR	NR
Iftikhar	2012	29.4 ± 7.77/28.3 ± 7.47	NR	80/652	652/652	NR	NR	80/309;73/343	NR	94(39–338)/94(68–208)
Joan	2006	NR	NR	NR	NR	NR	NR	NR	NR	NR
Lunde	2007	24.7(17,36.9)	NR	NR	NR	NR	NR	11	6	NR
Mani	2013	30.1 ± 7.6	NR	311/2,301	NR	130.5 ± 15.7	73.7 ± 11.1	NR	88/2,301	NR
Merz	2016	28.7 ± 5.9/30.0 ± 6.7	NR	9/46	All	141.4 ± 19.9/140.4 ± 21.6	75.4 ± 12.5/76.7 ± 10.9	12/171	6/87	109.78 ± 46.5/121.4 ± 60.5
Morgan	2012	28.7 ± 8.2/25.5 ± 5.8	NR	28,103/108670	NR	119.4 ± 14.3/116.8 ± 13.1	75.2 ± 14.3/72.5 ± 13.1	NR	713/21,734; 966/86,936	NR
Meun	2018	27.92 ± 4.53/26.84 ± 3.83	0.89 ± 0.08/0.86 ± 0.08	17/28	NR	142.3 ± 21.74/143.61 ± 19.22	74.55 ± 10.39/77.03 ± 9.92	70/107	20/12	6.25 ± 1.83/5.79 ± 1.41
Schmidt	2011	NR	NR	15/93	127/127	139.4 ± 20.2/123.1 ± 14.9	82.7 ± 10.6/79.1 ± 6.6	NR	NR	NR
Shaw	2008	31.1 ± 7/28.4 ± 6	0.885 ± 0.12/0.857 ± 0.11	27/45	390/390	139.9 ± 20/140.1 ± 22	77.4 ± 10/75.7 ± 11	63/104;126/286	34/104;70/286	132.1 ± 67/126.1 ± 58
Wild	2000	26.6/25.9	0.82/0.72	40/232	P/C:81/82	132/132	79/82	273/1,379	54/1,379	NR
**Study**	**Year**	**Insulin resistance, HOMA**	**Dyslipidemia**	**Total cholesterol**	**LDL cholesterol**	**HDL cholesterol**	**Triglycerides (mm/L)**	**Androstenedione (nmol/L)**	**Testosterone (mmol/L)**	**Free testosterone**
Calderon-Margalit	2014	5.32 ± 9.13/3.86 ± 2.96	8/48	4.90 ± 0.79/4.77 ± 0.87	2.79 ± 0.78/2.76 ± 0.76	1.55 ± 0.46/1.52 ± 0.41	1.22 ± 0.96/1.08 ± 0.79	NR	NR	NR
Dahlgren	1992	NR	NR	NR	NR	NR	40–49 Y: 1.16 ± 0.58/1.21 ± 0.52; 50–61 Y: 1.58 ± 0.92/1.35 ± 0.61	NR	NR	NR
Ding	2018	NR	NR	NR	NR	NR	NR	NR	NR	NR
Glintborg	2015	12.2(8.1–20.1)	112/108	1.0(0.7–1.5)	2.7(2.2–3.3)	1.4(1.1–1.6)	NR	NR	1.74(1.24–2.38)	0.033(0.021–0.050)
Hart	2015	NR	NR	NR	NR	NR	NR	NR	NR	NR
Iftikhar	2012	NR	MA	197(92–330)/199(0–369) mg/dl	108(26–225)/111(38–211) mg/dl	57(23–106)/58(23–129) mg/dl	107(29–473)/110 (33–431)	NR	NR	NR
Joan	2006	NR	NR	NR	NR	NR	NR	NR	NR	NR
Lunde	2007	NR	NR	NR	NR	NR	NR	NR	NR	NR
Mani	2013	NR	NR	NR	NR	NR	NR	NR	NR	NR
Merz	2016	3.07 ± 5.02/5.35 ± 8.24	16/146	195.3 ± 36.6/197.8 ± 48.5 mg/dl	110.1 ± 29.4/116.2 ± 42.0 mg/dl	47.9 ± 10.3/52.5 ± 11.2 mg/dl	NR	0.4	0.83	0.55
Morgan	2012	NR	NR	4.9 ± 1.0/4.9 ± 1.0 mmol/L	NR	NR	NR	NR	NR	NR
Meun	2018	NR	NR	5.98 ± 0.85/6.09 ± 1.01	NR	1.40 ± 0.35/1.57 ± 0.39	NR	2.63(2.00–3.31)/2.17(1.67–2.88)	1.30(1.04–1.71)/0.74(0.68–0.80)	2.69(2.13–3.49)/1.16(1.05–1.29)
Schmidt	2011	NR	MA	5.9 ± 0.8/5.9 ± 1.1 mmol/L	2.2 ± 0.7/2.7 ± 0.8 mmol/L	2.0 ± 0.4/1.6 ± 0.3 mmol/L	1.4 ± 0.7/1.0 ± 0.5	NR	NR	NR
Shaw	2008	35/104;63/286	NR	203.7 ± 49/194.4 ± 46 mg/dl	120.2 ± 41/114.4 ± 42 mg/dl	50.8 ± 12/52.4 ± 11 mg/dl	184.3 ± 147/147.0 ± 113	NR	NR	NR
Wild	2000	NR	NR	NR	NR	NR	NR	NR	NR	NR

*BMI, body mass index; NR, Not reported*.

### Results of Mate Analysis

#### All-Cause Mortality

**Figure 2:**
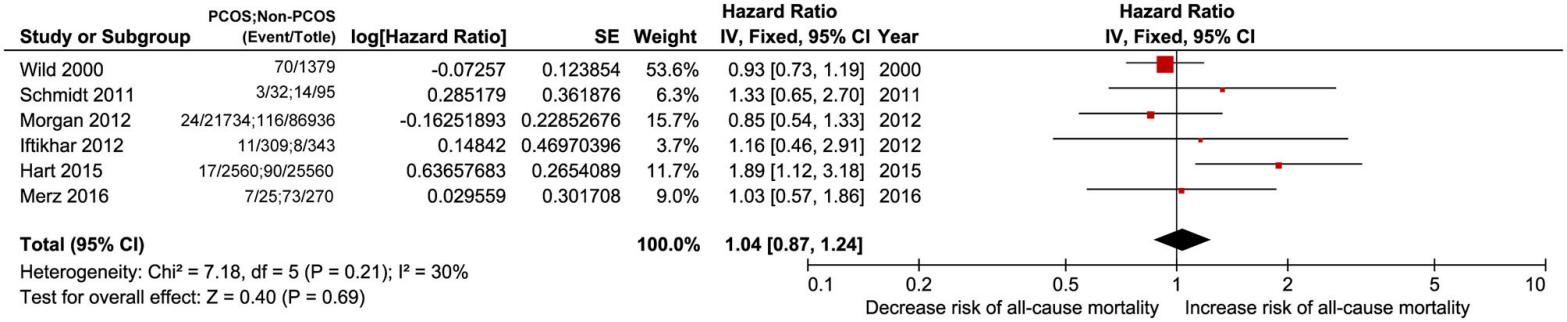
Forest plot comparing PCOS and non-PCOS for all-cause mortality.

Six studies including PCOS and non-PCOS were involved in the outcome of all-cause mortality evaluation. Compared with patients in the non-PCOS group, patients in the PCOS group had no significantly increased risk of all-cause mortality (HR: 1.04, 95% CI: 0.57–1.86, *P* = 0.69; *I*^2^ = 30%, *P* = 0.21) in a fixed-effects model (Figure 2).

#### Cardiovascular Death

**Figure 3:**
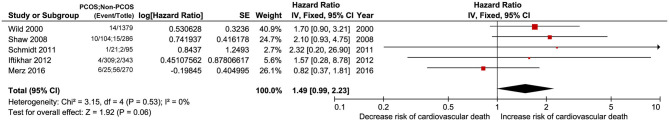
Forest plot comparing PCOS and non-PCOS for cardiovascular death.

Five studies including PCOS and non-PCOS were involved in the outcome of cardiovascular death evaluation. Compared with patients in the non-PCOS group, patients in the PCOS group had no significantly increased risk of cardiovascular death (HR: 1.49, 95% CI: 0.99–2.23, *P* = 0.06; *I*^2^ = 0%, *P* = 0.53) in a fixed-effects model (Figure 3).

#### Any CVD

**Figure 4:**
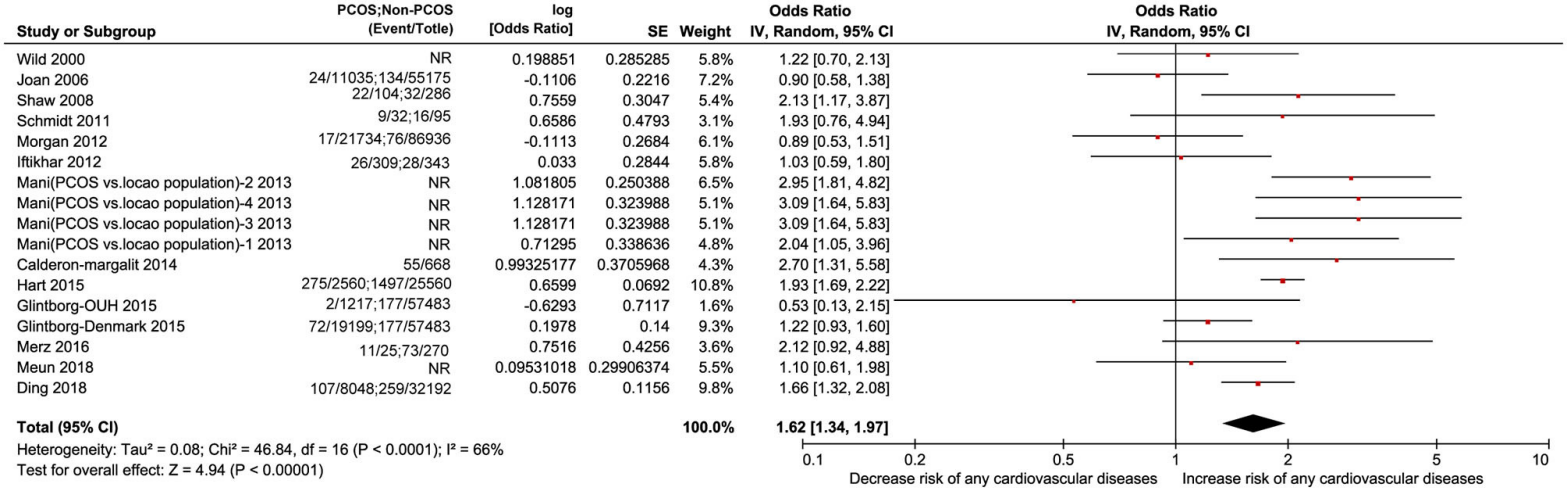
Forest plot comparing PCOS and non-PCOS for any CVDs. NR, not reported.

Seventeen studies including PCOS and non-PCOS were involved in the outcome of any CVD evaluation. Compared with patients in the non-PCOS group, patients in the PCOS group had significantly increased risk of CVD (OR: 1.66, 95% CI: 1.32–2.08, *P* < 0.00001; *I*^2^ = 66%, *P* < 0.0001) in a random-effects model (Figure 4).

#### MI

**Figure 5:**
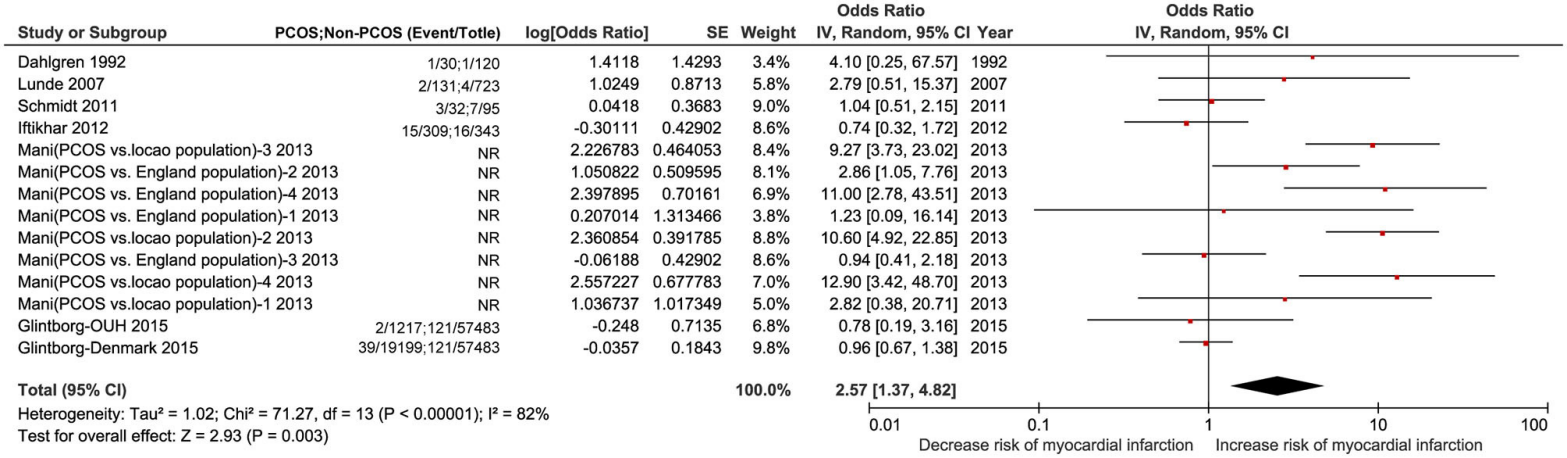
Forest plot comparing PCOS and non-PCOS for MI. NR, not reported.

Fourteen studies including PCOS and non-PCOS were involved in the outcome of MI evaluation. Compared with patients in the non-PCOS group, patients in the PCOS group had significantly increased risk of MI (OR: 2.57, 95% CI: 1.37–4.82, *P* = 0.003; *I*^2^ = 82%, *P* < 0.00001) in a random-effects model (Figure 5). There was larger heterogeneity in this subgroup analysis using the random effects model.

#### Ischemic Heart Disease

**Figure 6:**
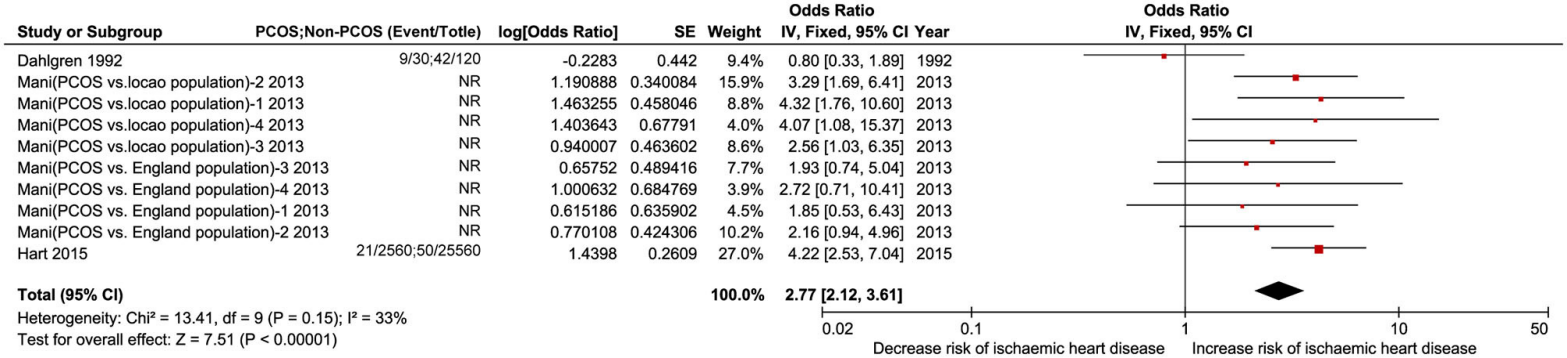
Forest plot comparing PCOS and non-PCOS for ischemic heart disease. NR, not reported.

Ten studies including PCOS and non-PCOS were involved in the outcomes of ischemic heart disease evaluation. Compared with patients in the non-PCOS group, patients in the PCOS group had significantly increased risk of ischemic heart disease (OR: 2.77, 95% CI: 2.12–3.61, *P* < 0.00001; *I*^2^ = 33%, *P* = 0.15) in a fixed-effects model (Figure 6). No heterogeneity was found in this subgroup analysis.

#### Stroke

**Figure 7:**
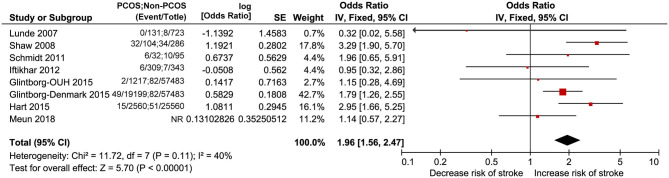
Forest plot comparing PCOS and non-PCOS for stroke.

Eight studies including PCOS and non-PCOS were involved in the outcome of stroke evaluation. Compared with patients in the non-PCOS group, patients in the PCOS group had significantly increased risk of stroke (OR: 1.96, 95% CI: 1.56–2.47, *P* < 0.00001; *I*^2^ = 40%, *P* = 0.11) in a fixed-effects model (Figure 7).

### Publication Bias

**Figure 8:**
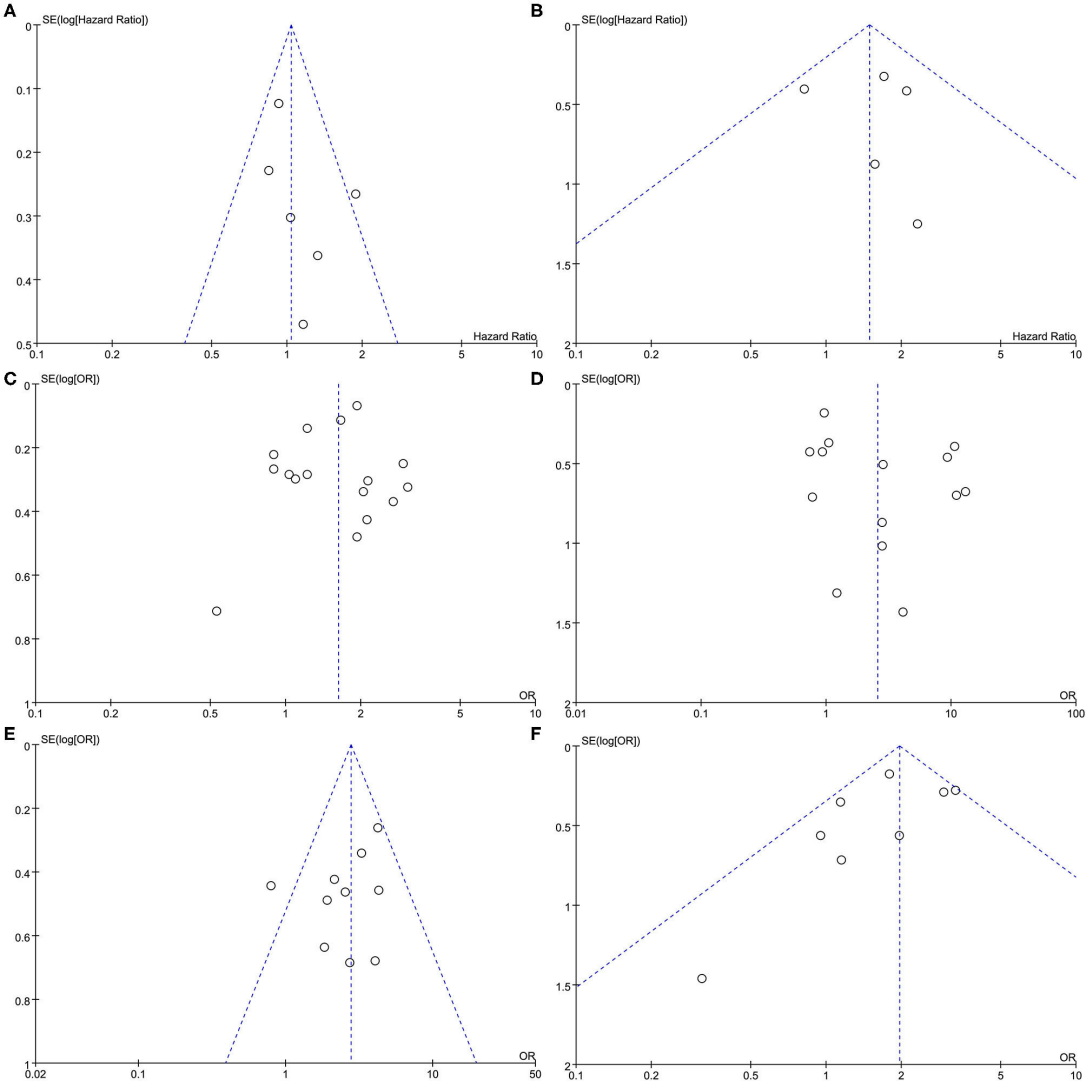
Funnel plot comparing PCOS and non-PCOS in all outcomes. **(A)** All cause morality. **(B)** Cardiovascular death. **(C)** Any cardiovascular diseases. **(D)** Myocardial infarction. **(E)** Ischemic heart diseases. **(F)** Stroke.

Funnel plots were performed for all outcomes presented in Figures 8A–F. Funnel plots of all outcomes were roughly symmetric, and no significant publication bias was found.

## Discussion

A large amount of data in this meta-analysis shows that PCOS and non-PCOS significantly differ in the risk of cardiovascular and cerebrovascular events, including any CVDs, MI, ischemic heart disease, and stroke, which indicates the relationship between PCOS and CVD risk. However, it did not lead to an increase in the associated mortality rate. To this day, the common pathophysiologic mechanisms are also unclear and require further study (8, 11).

In recent years, PCOS prevalence has gradually increased, and the clinical mechanism of its metabolism has received much attention (1, 33). Metabolic syndrome is a group of clinical syndromes characterized by the accumulation of various heart and cerebral risk factors, including hyperinsulinemia, IR causing obesity, hypertension, and dyslipidemia, and it directly promotes the atherosclerosis course, wherein CVD and CeVD are the main clinical consequences (34). PCOS prevalence is on the rise, which may be related to genetic factors, dyslipidemia, and hypertension combined with excessive androgen, which may explain the increased risk factor of CVD in patients with PCOS (18, 27, 35). The relative risk of MI in PCOS women increases four times between the ages of 40 and 49 and 11 times between the ages of 50 and 60 (36, 37). On the basis of a report from the American Heart Association in 2019 (38), accounting for 31% of the total number of deaths from CVD, it has become a common and frequently occurring disease that seriously affects human quality of life.

IR and hyperinsulinemia are common in PCOS (39). IR can induce and enhance oxidative stress, affect vascular endothelial function, cause vascular endothelial injury and decreased vascular compliance, and promote CVD progress (40). Changes in intrinsic risk mechanisms, including increased LDL and TG synthesis, enhanced gluconeogenics, decreased lipoprotein lipase activity, decreased liver clearance of LDL and TG, and abnormal lipid metabolism production, lead to obesity in the PCOS population (41). PCOS causes the liver to secrete LDL, and the conversion of cholesterol from HDL to LDL accelerates. HDL reduces and increases the risk of atherosclerosis, dyslipidemia (42). Moreover, PCOS patient's metabolic disturbances also cause hypertension that mainly changes vascular function as caused by hyperinsulinemia (43). High insulin levels enable increased NA^+^ reabsorption of the kidneys, increases aldosterone secretion, and decreases prostacyclin synthesis and vascular smooth muscle cell proliferation (44). Further, inflammatory cytokines and leptin levels were increased due to excessive fat accumulation, which leads to IR and induction of sympathetic nerve excitement, leading to hypertension (45).

The risk factors for CVD are complex, such as hypertension, smoke, diabetes, obesity, and metabolic synthesis character (38). There is still no obvious effective treatment for PCOS at present, and the focus is still on preventing complications, especially the occurrence and development of cardiovascular-related events (46). Further research is needed to improve the diagnostic process with aims to select specific treatment, customizing the therapy and lifestyle modifications (46).

This study has some advantages. First of all, this meta-analysis shows that PCOS can increase the risk of cardiovascular and cerebrovascular events, including any CVDs, MI, ischemic heart disease, and stroke, but the mortality-related outcomes cannot. Second, compared with previous studies, our conclusions are basically consistent with the research opinions of other meta-analyses (9, 10) on risk of cardiovascular and cerebrovascular events, but there were differences in mortality outcomes. The results of the previous two studies were not only relatively simple, but also not statistically significant in other studies. In addition, compared with previous research results, the results of this paper are more comprehensive and include a larger study population, which makes the research results more convincing and provides readers with more comprehensive information. In the end, the inclusion results of this paper are consistent with the expected analysis results, which provide some treatment ideas and medication plans for the diagnosis and treatment of clinical departments.

However, the limitations should also be considered in this study. At first, the occurrence and development of cardiovascular and cerebrovascular events in PCOS individuals are related to many factors, especially a series of physiological and biochemical indicators, such as BMI, blood glucose, and blood lipids. However, the original study did not provide too much relevant data for further analysis. This study cannot perform subgroup analysis and meta-regression to explore and resolve clinical heterogeneity issues caused by these significantly related factors.

This study demonstrates that women with PCOS have increased risk of cardiovascular and cerebrovascular events, including any CVDs, MI, ischemic heart disease, and stroke but not mortality-related outcomes. Clinicians should provide timely, effective, and personalized interventions to prevent or delay the occurrence and development of related reinsurance to improve the quality of life of patients with PCOS. Our study may require a larger sample size to further verify the conclusions.

## Data Availability Statement

All datasets generated for this study are included in the article/Supplementary Material.

## Author Contributions

This study was designed by J-HX. JZ and Q-QQ contributed data to the paper. Statistical analysis and interpretation of data were performed by J-HX, JZ and G-QZ. All authors were involved in drafting and revision of the manuscript for important intellectual content and approved the final version to be published.

## Conflict of Interest

The authors declare that the research was conducted in the absence of any commercial or financial relationships that could be construed as a potential conflict of interest.
