# Supplementary material for: Risk of Cardiovascular and Cerebrovascular Events in Polycystic Ovarian Syndrome Women: A Meta-Analysis of Cohort Studies
Source: Front Cardiovasc Med. 2020 Nov 12;7:552421. doi: 10.3389/fcvm.2020.552421 (PMC7690560; doi:10.3389/fcvm.2020.552421)
Supplement: Supplementary file 1 [file Table_1.DOCX]

**Supplementary Method 1** Search strategy

**1. Ovid MEDLINE(R) and Epub Ahead of Print, In-Process & Other Non-Indexed Citations, Daily, and Versions(R)** **<1946 to June 1, 2020>**

1. (Polycystic ovary syndrome).mp.
2. PCOS.mp.
3. (Sclerocystic ovarian degeneration).ti,ab.
4. (Stein-Leventhal syndrome).ti,ab.
5. #1 OR #2 OR #3 OR #4
6. Mortality.mp.
7. (Cardiovascular death).mp.
8. (Cardiovascular diseases).mp.
9. (Coronary heart disease).mp.
10. (Myocardial infarction).mp.
11. (Myocardial infarct).mp.
12. (Cardiovascular stroke).mp.
13. (Heart attack).mp.
14. (Myocardial Ischemia).mp.
15. Stroke.mp.
16. (Cerebrovascular accident).mp.
17. Apoplexy.mp.
18. #6 OR #7 OR #8 OR #9 OR #10 OR #11 OR #12 OR #13 OR #14 OR #15 OR #16 OR #17
19. #5 AND #18

**2. Ovid EMbase <1946 to June 1, 2020>**

1. (Polycystic ovary syndrome).mp.
2. PCOS.ti,ab.
3. (Sclerocystic ovarian degeneration).ti,ab.
4. (Stein-Leventhal syndrome).ti,ab.
5. #1 OR #2 OR #3 OR #4
6. Mortality.mp.
7. (Cardiovascular death).mp.
8. (Cardiovascular diseases).mp.
9. (Coronary heart disease).mp.
10. (Myocardial infarction).mp.
11. (Myocardial infarct).mp.
12. (Cardiovascular stroke).mp.
13. (Heart attack).mp.
14. (Myocardial Ischemia).mp.
15. Stroke.mp.
16. (Cerebrovascular accident).mp.
17. Apoplexy.mp.
18. #6 OR #7 OR #8 OR #9 OR #10 OR #11 OR #12 OR #13 OR #14 OR #15 OR #16 OR #17
19. #5 AND #18

**3. Web of Science (ISI) <1970 to June 1, 2020>**

1. Topic: (Polycystic ovary syndrome)

Databases= SCI-EXPANDED, SSCI, A&HCI, CPCI-S, CPCI-SSH, BKCI-S, BKCI-SSH, ESCI, CCR-EXPANDED, IC Timespan= 1970-2020

1. Topic: (PCOS)

Databases= SCI-EXPANDED, SSCI, A&HCI, CPCI-S, CPCI-SSH, BKCI-S, BKCI-SSH, ESCI, CCR-EXPANDED, IC Timespan= 1970-2020

1. Topic: (Stein-Leventhal syndrome)

Databases= SCI-EXPANDED, SSCI, A&HCI, CPCI-S, CPCI-SSH, BKCI-S, BKCI-SSH, ESCI, CCR-EXPANDED, IC Timespan= 1970-2020

1. Topic: (Sclerocystic ovarian degeneration)

Databases= SCI-EXPANDED, SSCI, A&HCI, CPCI-S, CPCI-SSH, BKCI-S, BKCI-SSH, ESCI, CCR-EXPANDED, IC Timespan= 1970-2020

1. #1 OR #2 OR #3 OR #4
2. Topic: (Mortality)

Databases= SCI-EXPANDED, SSCI, A&HCI, CPCI-S, CPCI-SSH, BKCI-S, BKCI-SSH, ESCI, CCR-EXPANDED, IC Timespan= 1970-2020

1. Topic: (Cardiovascular death)

Databases= SCI-EXPANDED, SSCI, A&HCI, CPCI-S, CPCI-SSH, BKCI-S, BKCI-SSH, ESCI, CCR-EXPANDED, IC Timespan= 1970-2020

1. Topic: (Cardiovascular diseases)

Databases= SCI-EXPANDED, SSCI, A&HCI, CPCI-S, CPCI-SSH, BKCI-S, BKCI-SSH, ESCI, CCR-EXPANDED, IC Timespan= 1970-2020

1. Topic: (Coronary heart disease)

Databases= SCI-EXPANDED, SSCI, A&HCI, CPCI-S, CPCI-SSH, BKCI-S, BKCI-SSH, ESCI, CCR-EXPANDED, IC Timespan= 1970-2020

1. Topic: (Myocardial infarction)

Databases= SCI-EXPANDED, SSCI, A&HCI, CPCI-S, CPCI-SSH, BKCI-S, BKCI-SSH, ESCI, CCR-EXPANDED, IC Timespan= 1970-2020

1. Topic: (Cardiovascular stroke)

Databases= SCI-EXPANDED, SSCI, A&HCI, CPCI-S, CPCI-SSH, BKCI-S, BKCI-SSH, ESCI, CCR-EXPANDED, IC Timespan= 1970-2020

1. Topic: (Myocardial infarct)

Databases= SCI-EXPANDED, SSCI, A&HCI, CPCI-S, CPCI-SSH, BKCI-S, BKCI-SSH, ESCI, CCR-EXPANDED, IC Timespan= 1970-2020

1. Topic: (Heart attack)

Databases= SCI-EXPANDED, SSCI, A&HCI, CPCI-S, CPCI-SSH, BKCI-S, BKCI-SSH, ESCI, CCR-EXPANDED, IC Timespan= 1970-2020

1. Topic: (Myocardial Ischemia)

Databases= SCI-EXPANDED, SSCI, A&HCI, CPCI-S, CPCI-SSH, BKCI-S, BKCI-SSH, ESCI, CCR-EXPANDED, IC Timespan= 1970-2020

1. Topic: (Stroke)

Databases= SCI-EXPANDED, SSCI, A&HCI, CPCI-S, CPCI-SSH, BKCI-S, BKCI-SSH, ESCI, CCR-EXPANDED, IC Timespan= 1970-2020

1. Topic: (Cerebrovascular accident)

Databases= SCI-EXPANDED, SSCI, A&HCI, CPCI-S, CPCI-SSH, BKCI-S, BKCI-SSH, ESCI, CCR-EXPANDED, IC Timespan= 1970-2020

1. Topic: (Apoplexy)

Databases= SCI-EXPANDED, SSCI, A&HCI, CPCI-S, CPCI-SSH, BKCI-S, BKCI-SSH, ESCI, CCR-EXPANDED, IC Timespan= 1970-2020

1. #6 OR #7 OR #8 OR #9 OR #10 OR #11 OR #12 OR #13 OR #14 OR #15 OR #16 OR #17
2. #5 AND #18
